# Supplementary material for: Mobility constraints in segregation models
Source: Sci Rep. 2023 Jul 26;13:12087. doi: 10.1038/s41598-023-38519-6 (PMC10372033; doi:10.1038/s41598-023-38519-6)
Supplement: Supplementary file 1 — Supplementary Information. [file 41598_2023_38519_MOESM1_ESM.pdf]

Mobility constraints in segregation models
Supplementary Information

Daniele Gambetta, Giovanni Mauro, Luca Pappalardo

Contents

I. Supplementary Notes 2
A. Supplementary Note 1: Random spatial distribution of relevance 2
B. Supplementary Note 2: Configurations of the mobility-constrained models 2
C. Supplementary Note 3: Segregation metrics 2
D. Supplementary Note 4: Variability of the results 3
E. Supplementary Note 5: Segregation in the centre and the periphery 3
F. Supplementary Note 6: Interplay of distance and relevance with $\beta > 0$ 3
G. Supplementary Note 7: Critical point of centre segregation 3

II. Supplementary Figures 5

I. SUPPLEMENTARY NOTES

A. Supplementary Note 1: Random spatial distribution of relevance

We also explore model versions that incorporate a uniformly random spatial relevance distribution. However, as shown in Figure S1, the influence of α on the convergence time n is less prominent in this case. We still observe an exponential relationship, but the maximum value of n is reduced to 70 when $\alpha = 3$, compared to 260 when assuming a core-periphery relevance distribution. Similarly, the impact on the segregation level S is barely noticeable, with a minimal upward trend. In summary, adopting a uniformly random spatial distribution of relevance sacrifices realism and leads to less intriguing emerging patterns.

B. Supplementary Note 2: Configurations of the mobility-constrained models

In addition to the configuration discussed in the manuscript, we conducted additional experiments by varying the initial model setups (grid size, majority-minority ratio, homophily percentage, relocation policy) and using employing a different agent movement policy.

Grid size. We conduct experiments on two additional grid configurations: a smaller grid size of 25×25 and a larger grid size of 75×75. Figure S2 illustrates the impact of distance and relevance exponents on segregation dynamics. The findings align with those presented in the manuscript, although in the smaller grid, the correlations between variables are relatively weaker compared to the larger grids.

Homophily. We also investigate the impact of different levels of homophily (0.1 and 0.5) on segregation dynamics, in comparison to the homophily level discussed in the manuscript. Figure S3 presents the results of the experiments examining the effects of distance and relevance exponents (β and α). Regarding the influence of β, both cases align with the findings in the manuscript, showing similar conclusions. In the relevance model, when the homophily level is set to 0.5, the same conclusions as the manuscript are obtained. However, when the homophily level is reduced to 0.1, the convergence time n becomes low for all values of α (less than 5). As a result, the variation of n with changing α becomes insignificant. This outcome can be attributed to the fact that with low homophily, agents can easily find cells where they are happy, leading to faster convergence.

Population distribution. We examined the impact of different population distributions between minority and majority agents: a less even distribution (10%/90%) and a more balanced distribution (50%/50%). In the case of the 10%/90% distribution, we also set the density to 0.5. This adjustment was necessary because having a very high majority population led to persistent unhappiness in the minority, preventing the models from converging within 500 steps. Consequently, the analysis would have been less informative. Figure S6 an S7 displays the results of the experiments investigating the effects of distance and relevance exponents on segregation dynamics. Regarding the influence of β, both population distribution cases yielded the same conclusions as the manuscript. In the relevance model, when the distribution was 50%/50%, the same conclusions as the manuscript were observed. However, when the distribution was 10%/90%, the convergence time n was very low for all values of α (less than 5). As a result, the variation of n with changing α became insignificant. This can be attributed to the fact that in the 10%/90% distribution, agents easily found cells where they were happy, leading to faster convergence.

Relocation policy. In the experiments conducted in the manuscript, we implement a movement policy where, at each step, unhappy agents moved to a random empty cell. In this section, we repeat the experiment with a modified movement policy. Instead of selecting a random empty cell, we only consider empty cells where an agent would be happy. Figure S8 presents the results of these experiments, illustrating the effects of distance and relevance exponents on segregation dynamics using the improved movement policy. In general, although the results are in line with those in the manuscript, the convergence time n observed is lower compared to the manuscript because agents have a higher probability of reaching a happy configuration.

C. Supplementary Note 3: Segregation metrics

In the manuscript, we employed a classic index to measure segregation. This index was calculated by determining the average number of similar neighbors and dividing it by the total number of neighbors. An increasing value of this index indicates a greater level of segregation. In order to delve deeper into the dynamics of segregation, we conducted supplementary experiments employing an alternative measure known as the generalized Freeman Segregation Index (FSI). The FSI takes into account the presence of cross-links, which represent connections between nodes or agents belonging to different categories or types. Here, we compute the number of cross-links, denoted as X_n, at each step. This quantity is obtained by summing the number of contacts between agents of different types in immediate

proximity. Subsequently, we calculate the FSI at each step, denoted as FSI_n, by dividing the count of cross-links at that step by the count of cross-links at the beginning of the simulation (step 0). Mathematically, it can be expressed as:

$$FSI_n = \frac{|X_n|}{|X_0|}$$

It is worth noting that in this case, as the simulation's degree of segregation increases, the FSI values, which initially start at 1 in the first step, will decrease. This occurs because the number of neighbors of a different type diminishes as segregation intensifies. Figure S9 demonstrates that these experiments yield the same conclusions as those presented in the manuscript. The observed trends (reversed in nature, given that an higher FSI signifies a less segregated scenario) and patterns align with the findings discussed earlier, further validating the results and reinforcing the main conclusions of the study.

D. Supplementary Note 4: Variability of the results

Figure S10 shows the standard deviation around the average of S and n as β and α vary, showing consistency and small variations. Indeed, despite these variations, the fundamental patterns and trends described in the main paper remain clearly visible. In Figure S11, we present the distribution of the segregation level S and the simulation time n for each combination of β and α values. The distributions exhibit peaks in all cases, indicating a concentrated range of values. However, for high values of β and α, there is a peak around 500 for the simulation time n. This is primarily because, in these instances, the simulation often fails to converge within the 500 simulation steps (the maximum number of steps allowed).

E. Supplementary Note 5: Segregation in the centre and the periphery

As mentioned in the manuscript, increasing the parameter α tends to amplify the difference in segregation levels between the periphery and the center, as represented by $S_{\text{diff}} = S_{\text{periphery}} - S_{\text{centre}}$. This indicates that agents in the periphery exhibit a higher degree of segregation compared to those in the center, resulting in a more segregated final grid (refer to Figure S12). Notably, when α reaches its maximum value ($\alpha = 3$), and β has a significant impact ($\beta \in [-5, -3]$), the highest S_{diff} is observed. It is noteworthy that the difference becomes almost negligible when $\alpha = 0$.

F. Supplementary Note 6: Interplay of distance and relevance with $\beta > 0$

In the manuscript, we focused on the case where $\beta < 0$, representing the cost of long travel for unhappy agents, and examined the segregation dynamics that arise in conjunction with the relevance parameter. However, for the sake of completeness, we also analyze the case when $\beta > 0$, which assigns higher probabilities to cells farther away from the location of unhappy agents. Figure S13 presents heatmaps illustrating the values of S and n for different β values ranging from -5 to 5. In the case of positive β values, we do not observe any significant evidence of a relationship between S and α. The segregation level, as represented by S, does not exhibit distinct patterns or trends corresponding to varying values of α. However, we do find that the convergence time n remains consistently low for positive β values. Unlike the case of negative β, where n varied significantly with different α values, the impact of α on n is not pronounced when $\beta > 0$. Despite the low convergence time, we do not observe substantial variations in n across different α values in this scenario. These findings highlight the contrasting dynamics between positive and negative values of β and underscore the role of the distance parameter in shaping segregation patterns.

G. Supplementary Note 7: Critical point of centre segregation

As highlighted in the manuscript, the simultaneous presence of significant values for both the relevance and distance exponents results in a notable increase in the convergence time n. This configuration gives rise to an interesting phenomenon in the segregation dynamics of the centre, resembling a phase transition, as shown in Figure S14 (with the same parameter configuration as in the manuscript). Specifically, the figure displays an initial sharp shift, indicating a transition from low to high levels of segregation in the centre. This transition is followed by an oscillation phase

where the segregation value of the centre fluctuates around 0.68. The red line represents the tipping point, which is identified as the step at which the increase in segregation value no longer exceeds 2% compared to the previous five steps. To gain further insights into the elongation of the convergence time n, we examine the distribution of unhappiness steps for each agent. We observe that the elongation is primarily caused by a few minority agents that remain unhappy for an extended period. These agents, referred to as persistently unhappy (p.u.) agents, are identified based on exceeding the 95th percentile in the distribution of steps to happiness. Remarkably, we find that these p.u. agents are predominantly located in an area known as suburbia during the tipping point step mentioned earlier. This observation holds a higher probability compared to other models, shedding light on the specific spatial patterns contributing to the prolonged convergence time in the simulation.

II. SUPPLEMENTARY FIGURES

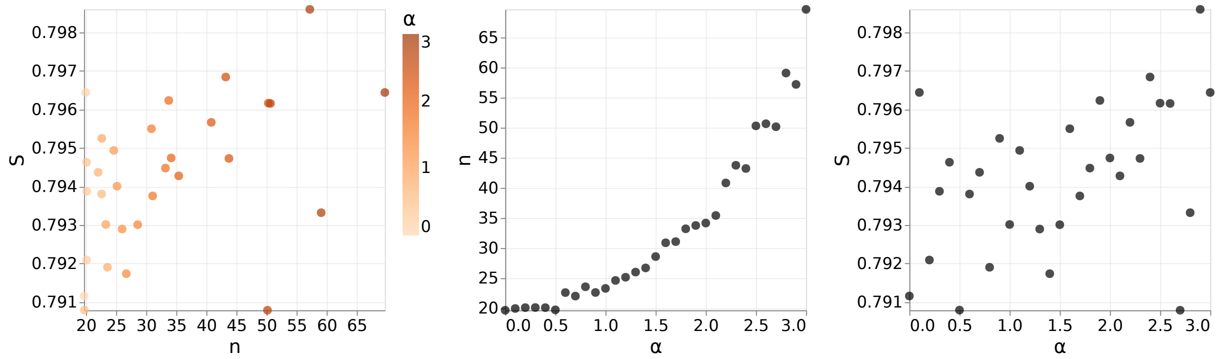

Figure S1: a) The average value of n and S over 100 simulations with the same value of α but different initial grid configurations, colour-coded by the value of α, considering a random distribution of relevance. Increasing values of α elongate n and slightly increase S (b) α vs average n over 100 simulations. (c) α vs average S over 100 simulations.

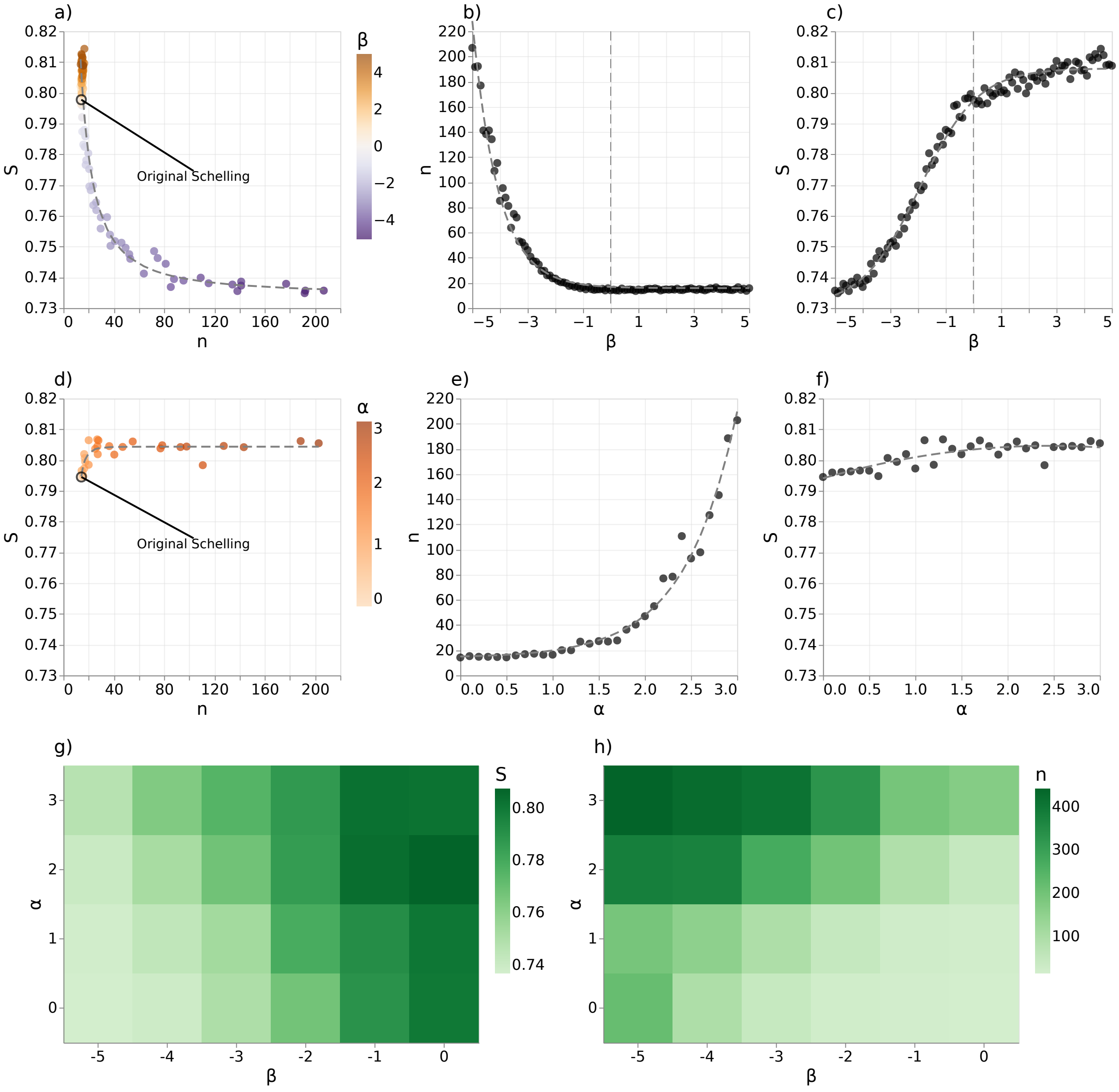

Figure S2: **Effects of distance and relevance exponents on segregation dynamics on a 25x25 grid** (a-c) Effects of β on segregation dynamics. (a) The average value of n and S over 100 simulations with the same β value but different initial grid configurations, colour-coded by the value of β. The lower β, the higher the cost of relocating far away, resulting in longer convergence time and reduced segregation levels compared to the original Schelling model. (b) β vs average n over 100 simulations. The lower β (< 0), the longer the simulation. (c) β vs average S over 100 simulations. For $\beta < 0$, there is an exponential increase in S; $\beta > 0$, the growth is moderate. (d-f) Effects of α on segregation dynamics. (d) The average value of n and S over 100 simulations with the same value of α but different initial grid configurations, colour-coded by the value of α. Increasing values of α elongate n and slightly increase S. (e) α vs average n over 100 simulations. (f) α vs average S over 100 simulations. (g) The average S (colour) for each combination of α and $\beta < 0$. For every value of α, higher β values lead to a higher S; for every β, higher α values lead to a higher S. (h) The average n (colour) for each combination of α and $\beta < 0$. For every value of α, higher β values lead to a lower n; for every β, higher α values lead to higher n.

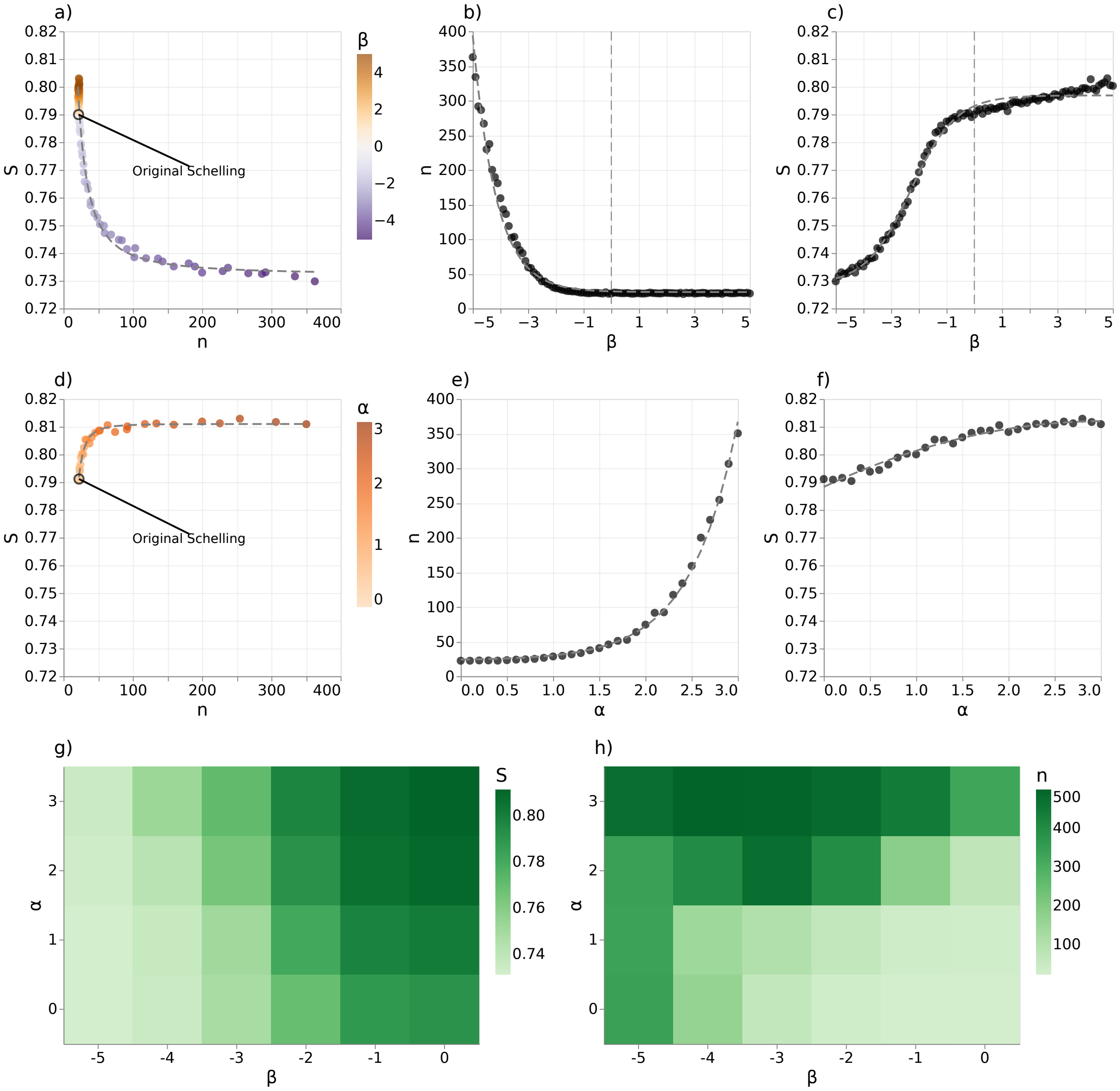

Figure S3: **Effects of distance and relevance exponents on segregation dynamics on a 75x75 grid** (a-c) Effects of β on segregation dynamics. (a) The average value of n and S over 100 simulations with the same β value but different initial grid configurations, colour-coded by the value of β. The lower β, the higher the cost of relocating far away, resulting in longer convergence time and reduced segregation levels compared to the original Schelling model. (b) β vs average n over 100 simulations. The lower β (< 0), the longer the simulation. (c) β vs average S over 100 simulations. For $\beta < 0$, there is an exponential increase in S; $\beta > 0$, the growth is moderate. (d-f) Effects of α on segregation dynamics. (d) The average value of n and S over 100 simulations with the same value of α but different initial grid configurations, colour-coded by the value of α. Increasing values of α elongate n and slightly increase S. (e) α vs average n over 100 simulations. (f) α vs average S over 100 simulations. (g) The average S (colour) for each combination of α and $\beta < 0$. For every value of α, higher β values lead to a higher S; for every β, higher α values lead to a higher S. (h) The average n (colour) for each combination of α and $\beta < 0$. For every value of α, higher β values lead to a lower n; for every β, higher α values lead to higher n.

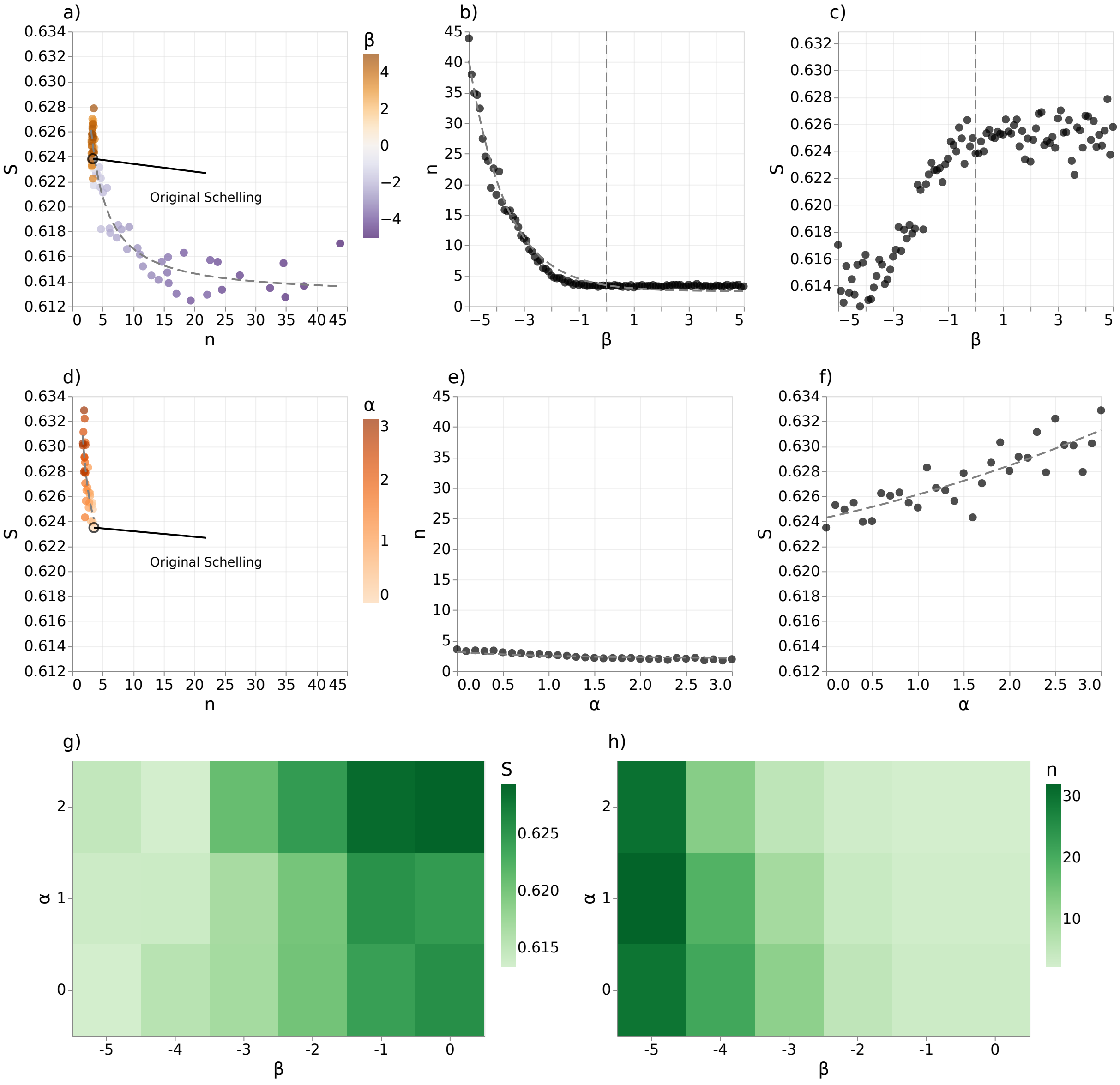

Figure S4: **Effects of distance and relevance exponents on segregation dynamics with homophily=0.1** (a-c) Effects of β on segregation dynamics. (a) The average value of n and S over 100 simulations with the same β value but different initial grid configurations, colour-coded by the value of β. The lower β, the higher the cost of relocating far away, resulting in longer convergence time and reduced segregation levels compared to the original Schelling model. (b) β vs average n over 100 simulations. The lower β (< 0), the longer the simulation. (c) β vs average S over 100 simulations. For $\beta < 0$, there is an exponential increase in S; $\beta > 0$, the growth is moderate. (d-f) Effects of α on segregation dynamics. (d) The average value of n and S over 100 simulations with the same value of α but different initial grid configurations, colour-coded by the value of α. Increasing values of α elongate n and slightly increase S. (e) α vs average n over 100 simulations. (f) α vs average S over 100 simulations. (g) The average S (colour) for each combination of α and $\beta < 0$. For every value of α, higher β values lead to a higher S; for every β, higher α values lead to a higher S. (h) The average n (colour) for each combination of α and $\beta < 0$. For every value of α, higher β values lead to a lower n; for every β, higher α values lead to higher n.

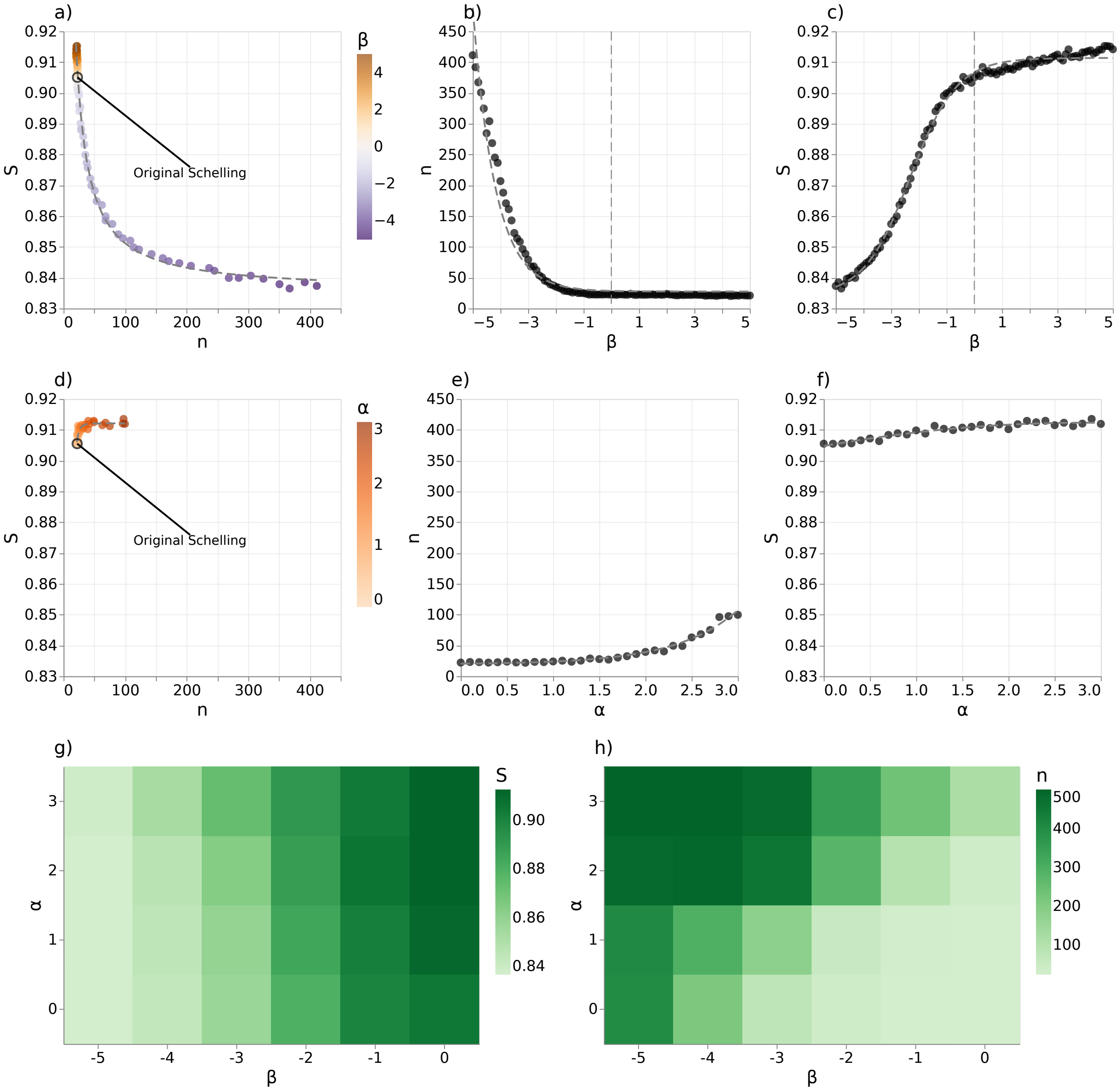

Figure S5: **Effects of distance and relevance exponents on segregation dynamics with homophily=0.5** (a-c) Effects of β on segregation dynamics. (a) The average value of n and S over 100 simulations with the same β value but different initial grid configurations, colour-coded by the value of β. The lower β, the higher the cost of relocating far away, resulting in longer convergence time and reduced segregation levels compared to the original Schelling model. (b) β vs average n over 100 simulations. The lower β (< 0), the longer the simulation. (c) β vs average S over 100 simulations. For $\beta < 0$, there is an exponential increase in S; $\beta > 0$, the growth is moderate. (d-f) Effects of α on segregation dynamics. (d) The average value of n and S over 100 simulations with the same value of α but different initial grid configurations, colour-coded by the value of α. Increasing values of α elongate n and slightly increase S. (e) α vs average n over 100 simulations. (f) α vs average S over 100 simulations. (g) The average S (colour) for each combination of α and $\beta < 0$. For every value of α, higher β values lead to a higher S; for every β, higher α values lead to a higher S. (h) The average n (colour) for each combination of α and $\beta < 0$. For every value of α, higher β values lead to a lower n; for every β, higher α values lead to higher n.

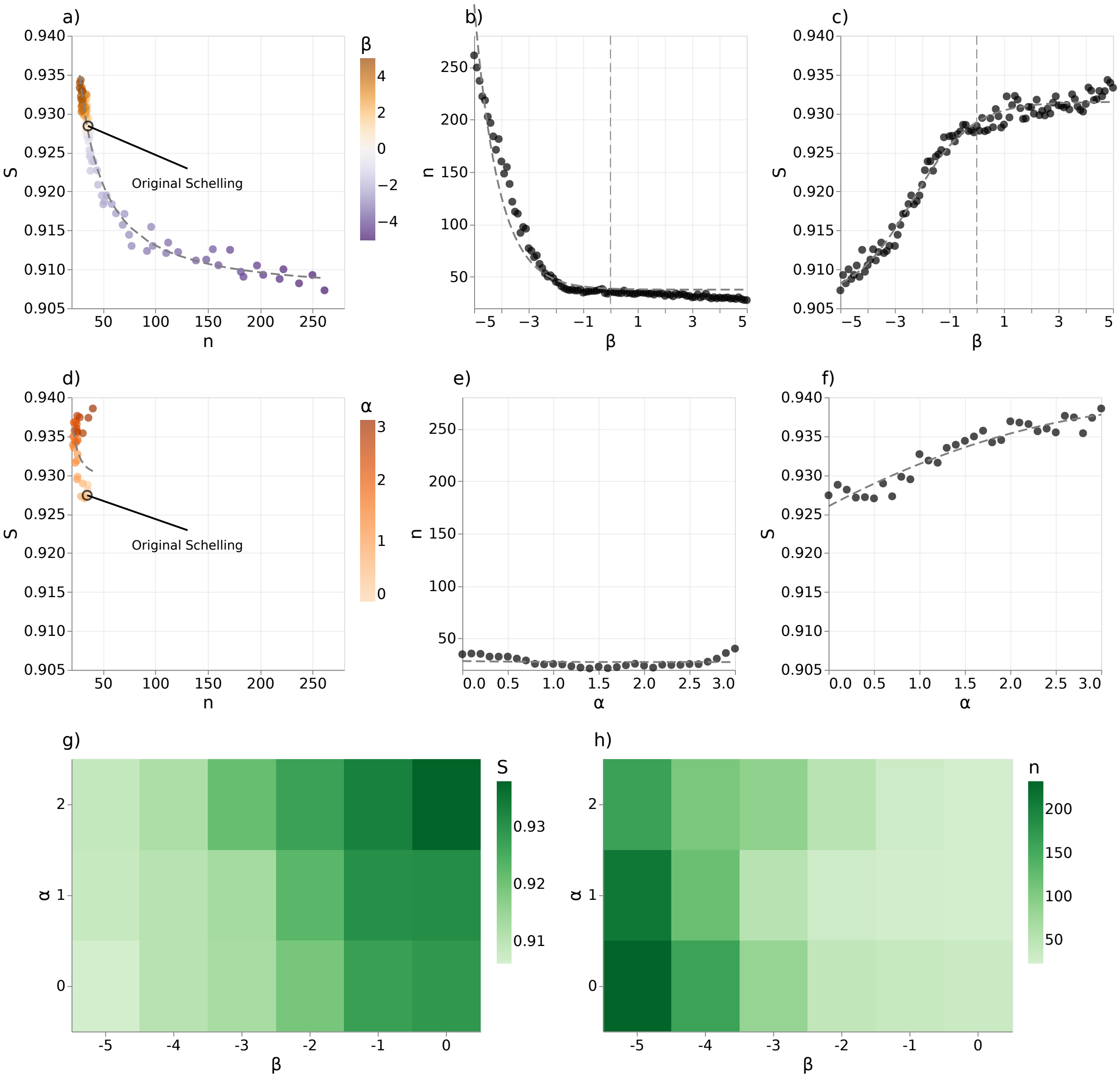

Figure S6: **Effects of distance and relevance exponents on segregation dynamics with distribution 10%/90% and density 0.5** (a-c) Effects of β on segregation dynamics. (a) The average value of n and S over 100 simulations with the same β value but different initial grid configurations, colour-coded by the value of β. The lower β, the higher the cost of relocating far away, resulting in longer convergence time and reduced segregation levels compared to the original Schelling model. (b) β vs average n over 100 simulations. The lower β (< 0), the longer the simulation. (c) β vs average S over 100 simulations. For $\beta < 0$, there is an exponential increase in S; $\beta > 0$, the growth is moderate. (d-f) Effects of α on segregation dynamics. (d) The average value of n and S over 100 simulations with the same value of α but different initial grid configurations, colour-coded by the value of α. Increasing values of α elongate n and slightly increase S. (e) α vs average n over 100 simulations. (f) α vs average S over 100 simulations. (g) The average S (colour) for each combination of α and $\beta < 0$. For every value of α, higher β values lead to a higher S; for every β, higher α values lead to a higher S. (h) The average n (colour) for each combination of α and $\beta < 0$. For every value of α, higher β values lead to a lower n; for every β, higher α values lead to higher n.

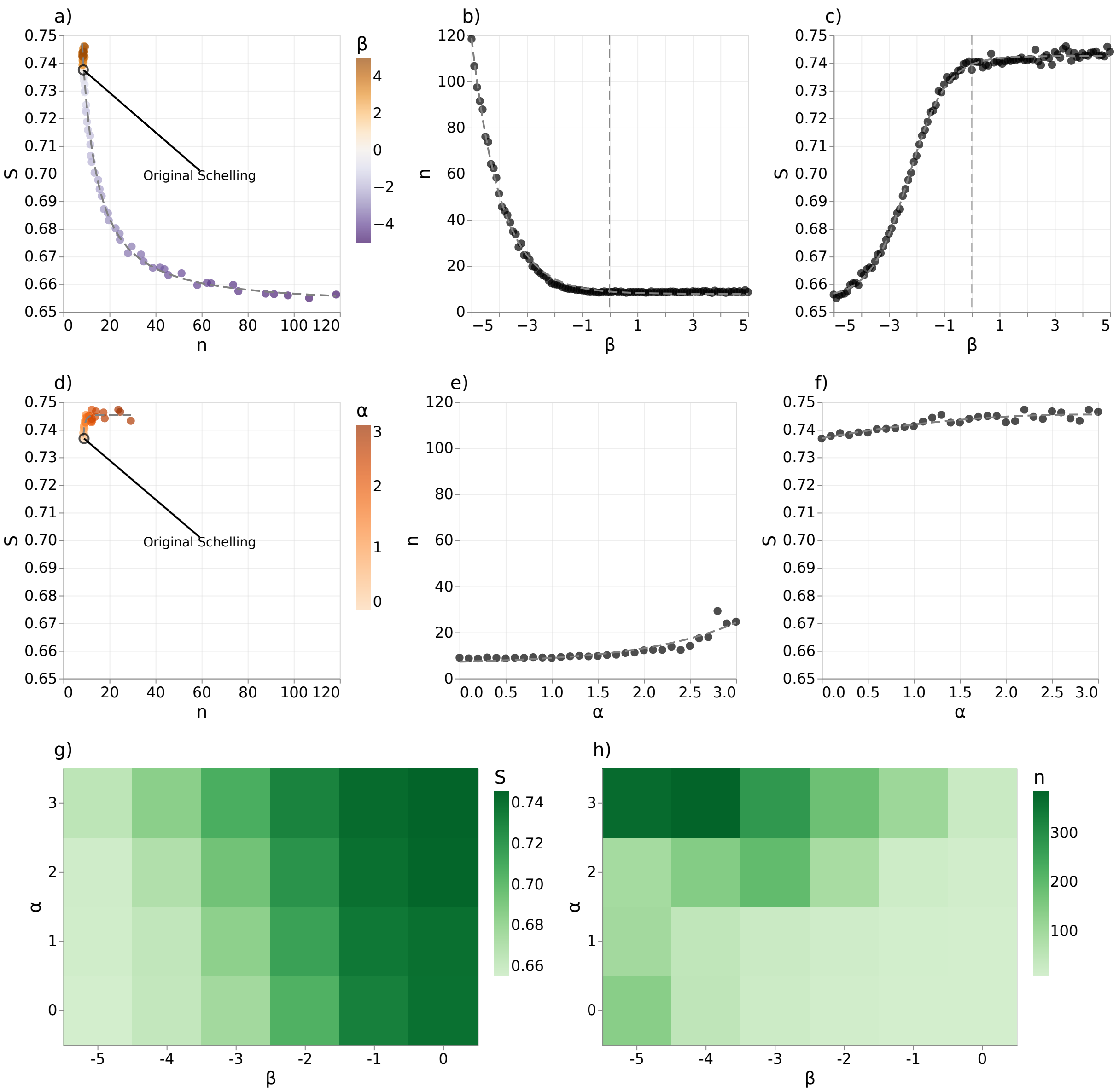

Figure S7: **Effects of distance and relevance exponents on segregation dynamics with distribution of population 50%/50%** (a-c) Effects of β on segregation dynamics. (a) The average value of n and S over 100 simulations with the same β value but different initial grid configurations, colour-coded by the value of β. The lower β, the higher the cost of relocating far away, resulting in longer convergence time and reduced segregation levels compared to the original Schelling model. (b) β vs average n over 100 simulations. The lower β (< 0), the longer the simulation. (c) β vs average S over 100 simulations. For $\beta < 0$, there is an exponential increase in S; $\beta > 0$, the growth is moderate. (d-f) Effects of α on segregation dynamics. (d) The average value of n and S over 100 simulations with the same value of α but different initial grid configurations, colour-coded by the value of α. Increasing values of α elongate n and slightly increase S. (e) α vs average n over 100 simulations. (f) α vs average S over 100 simulations. (g) The average S (colour) for each combination of α and $\beta < 0$. For every value of α, higher β values lead to a higher S; for every β, higher α values lead to a higher S. (h) The average n (colour) for each combination of α and $\beta < 0$. For every value of α, higher β values lead to a lower n; for every β, higher α values lead to higher n.

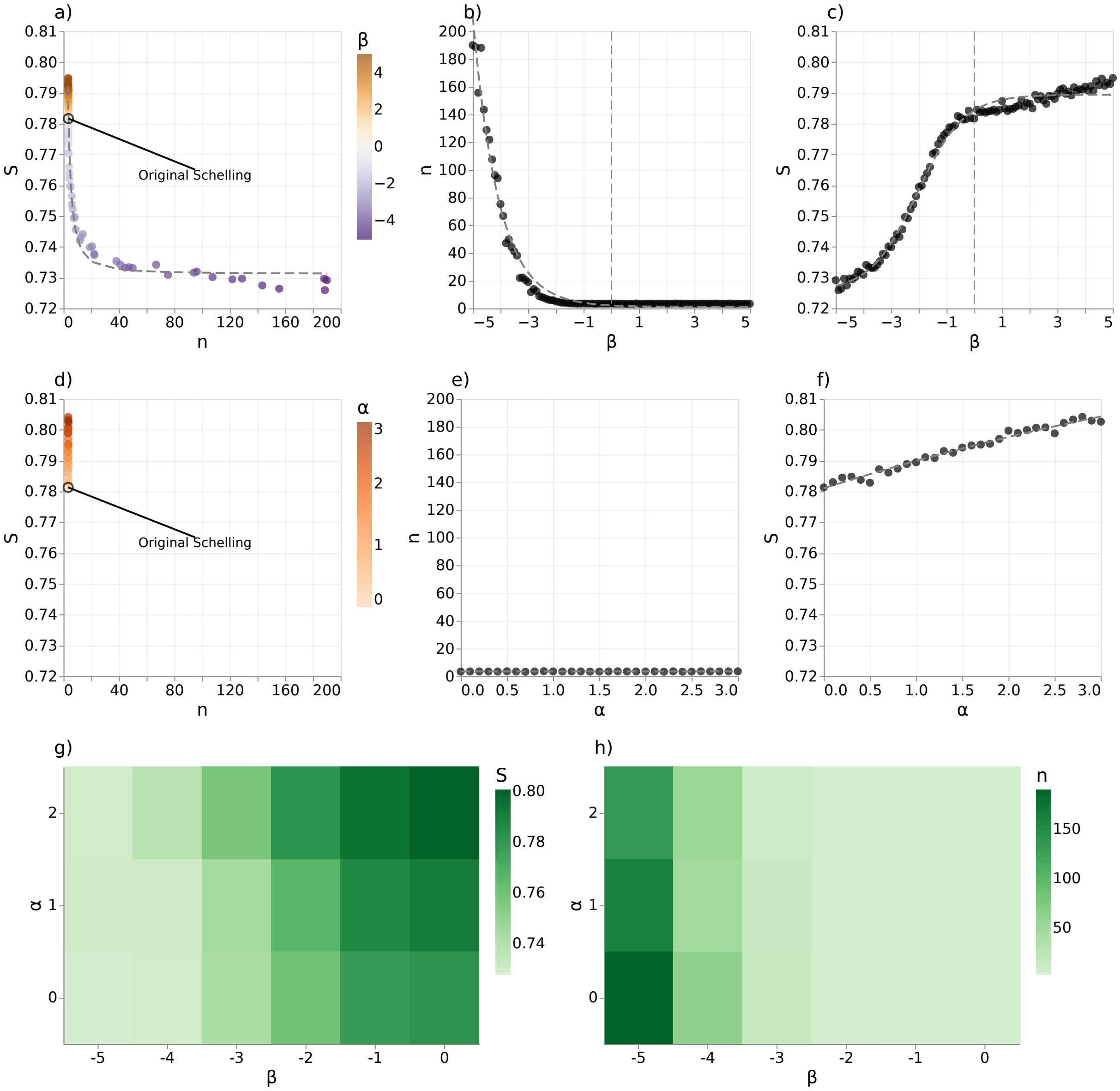

Figure S8: **Effects of distance and relevance exponents on segregation dynamics with Best policy** (a-c) Effects of β on segregation dynamics. (a) The average value of n and S over 100 simulations with the same β value but different initial grid configurations, colour-coded by the value of β. The lower β, the higher the cost of relocating far away, resulting in longer convergence time and reduced segregation levels compared to the original Schelling model. (b) β vs average n over 100 simulations. The lower β (< 0), the longer the simulation. (c) β vs average S over 100 simulations. For $\beta < 0$, there is an exponential increase in S; $\beta > 0$, the growth is moderate. (d-f) Effects of α on segregation dynamics. (d) The average value of n and S over 100 simulations with the same value of α but different initial grid configurations, colour-coded by the value of α. Increasing values of α elongate n and slightly increase S. (e) α vs average n over 100 simulations. (f) α vs average S over 100 simulations. (g) The average S (colour) for each combination of α and $\beta < 0$. For every value of α, higher β values lead to a higher S; for every β, higher α values lead to a higher S. (h) The average n (colour) for each combination of α and $\beta < 0$. For every value of α, higher β values lead to a lower n; for every β, higher α values lead to higher n.

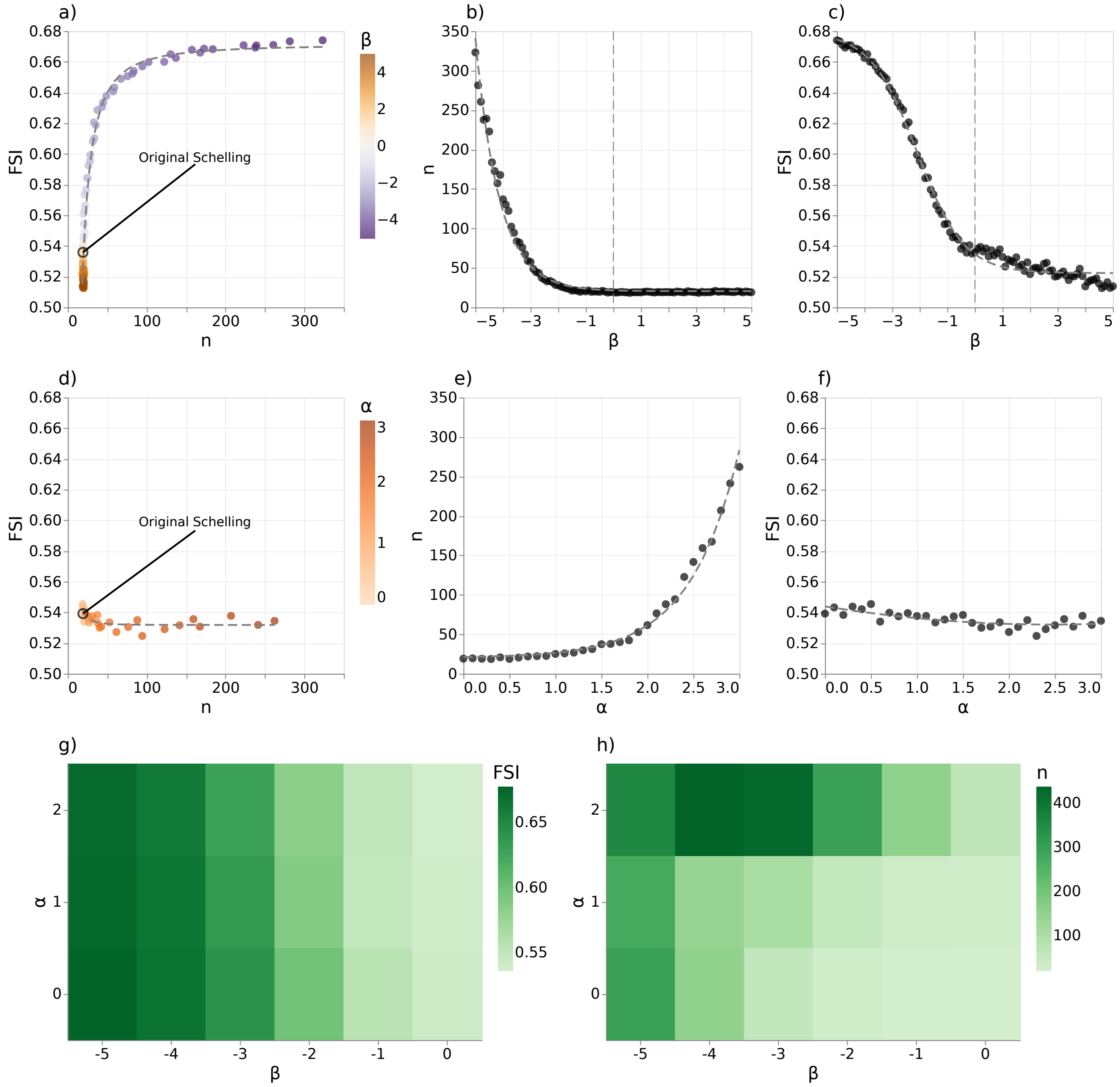

Figure S9: **Effects of distance and relevance exponents on segregation dynamics using FSI** (a-c) Effects of β on segregation dynamics. (a) The average value of n and FSI over 100 simulations with the same β value but different initial grid configurations, colour-coded by the value of β. The lower β, the higher the cost of relocating far away, resulting in longer convergence time and higher FSI compared to the original Schelling model. (b) β vs average n over 100 simulations. The lower β (< 0), the longer the simulation. (c) β vs average FSI over 100 simulations. For $\beta < 0$, there is an exponential decrease in FSI; $\beta > 0$, the growth is more moderate. (d-f) Effects of α on segregation dynamics. (d) The average value of n and FSI over 100 simulations with the same value of α but different initial grid configurations, colour-coded by the value of α. Increasing values of α elongate n and slightly decrease FSI. (e) α vs average n over 100 simulations. (f) α vs average FSI over 100 simulations. (g) The average FSI (colour) for each combination of α and $\beta < 0$. For every value of α, higher β values lead to a lower FSI; for every β, higher α values lead to a a slightly lower FSI. (h) The average n (colour) for each combination of α and $\beta < 0$. For every value of α, higher β values lead to a lower n; for every β, higher α values lead to higher n.

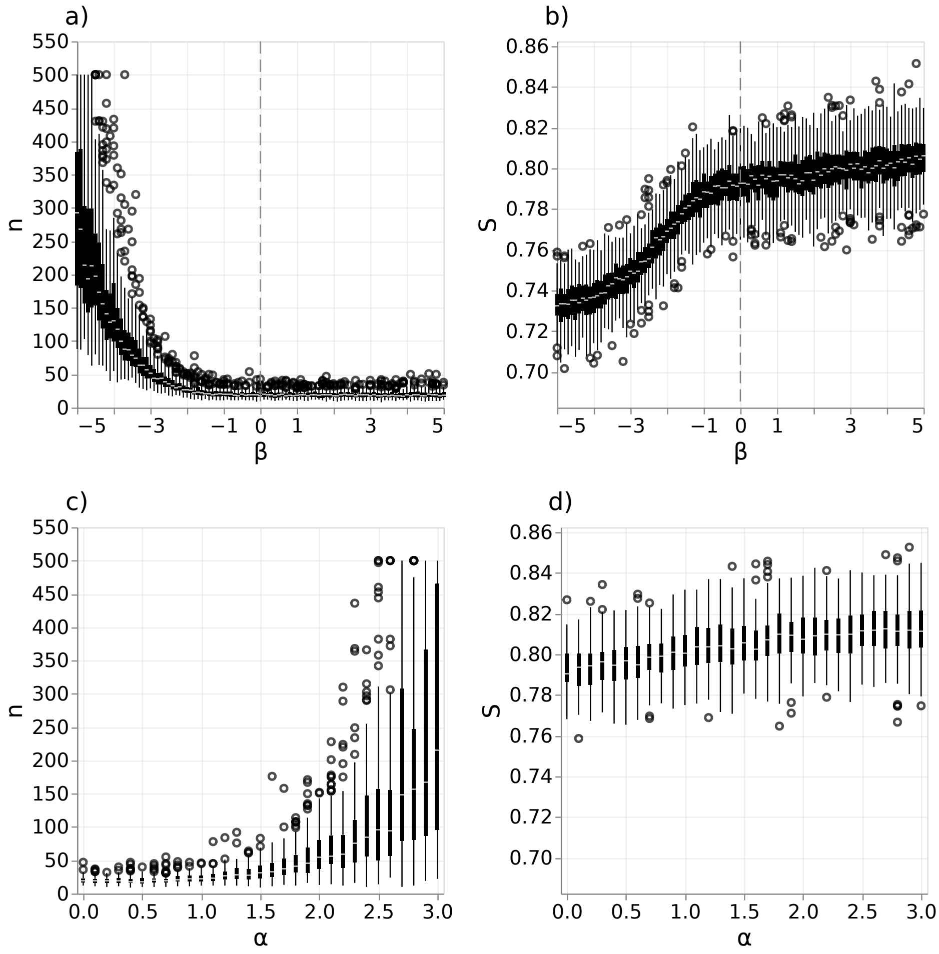

Figure S10: Boxplots representing the distribution of n and S over 100 simulations as β and α varies.

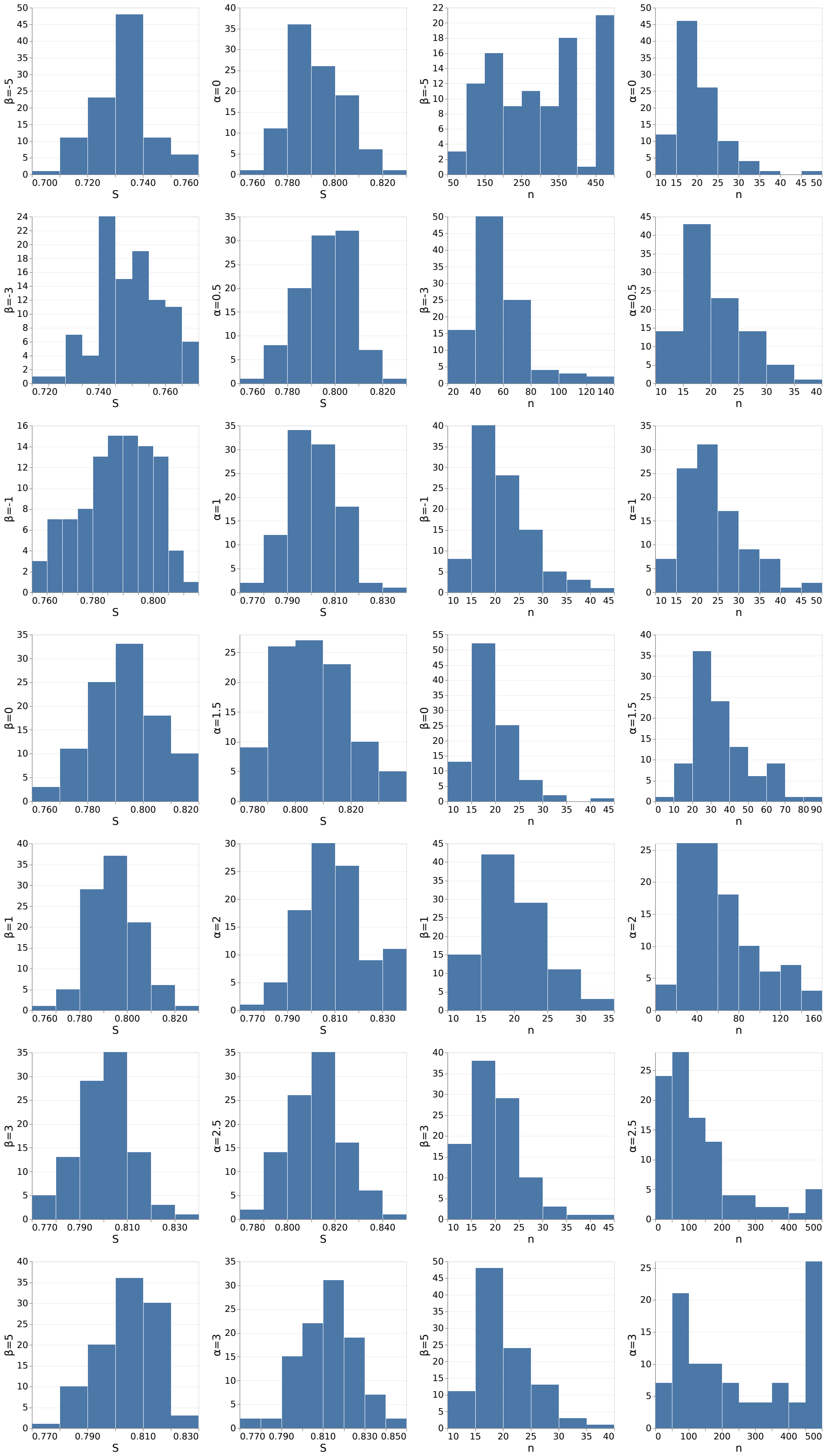

Figure S11: Distribution of S and n over 100 simulations as β and α varies. The distributions exhibit peaks in all cases, indicating a concentrated range of values. However, for high values of both β and α, there is a peak around 500 for the simulation time n due to the fact that, in these instances, the simulation fails to converge within the designated 500 simulation steps.

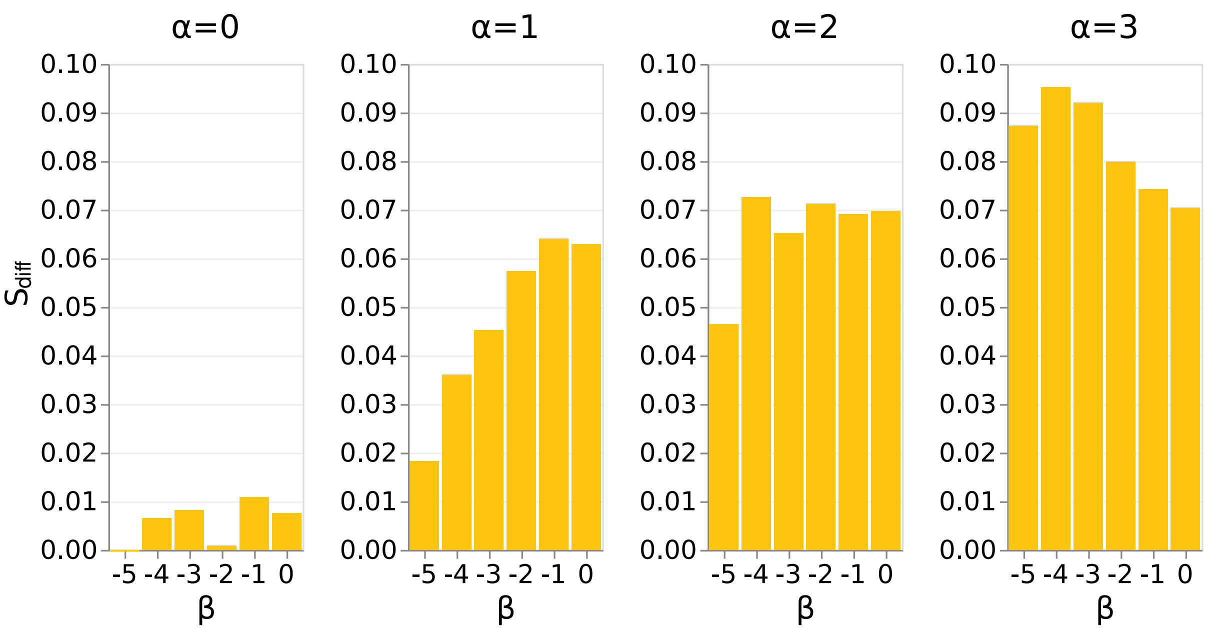

Figure S12: Each plot shows the difference between segregation of the periphery and segregation of center $S_{\mathrm{diff}} = S_{\mathrm{periphery}} - S_{\mathrm{centre}}$ for combinations of values of α and β. Higher values of α lead to increase of S_{diff}.

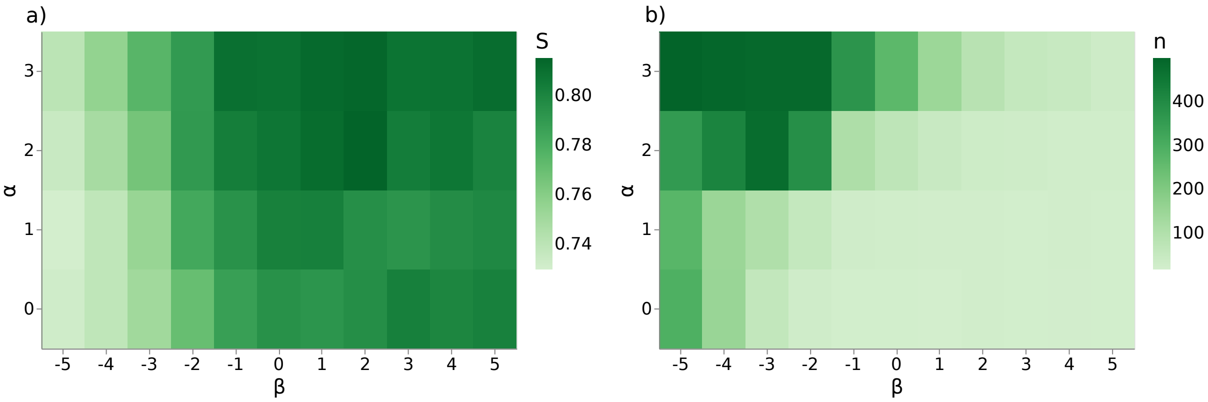

Figure S13: (a) The average S (colour) for each combination of α and β also with $\beta > 0$. For positive values of β, S doesn't show particular evidence of relation with α. (b) The average n (colour) for each combination of α and β also with $\beta > 0$. For positive values of β, n remains low but doesn't show particular variation among different values of α like in case of $\beta < 0$.

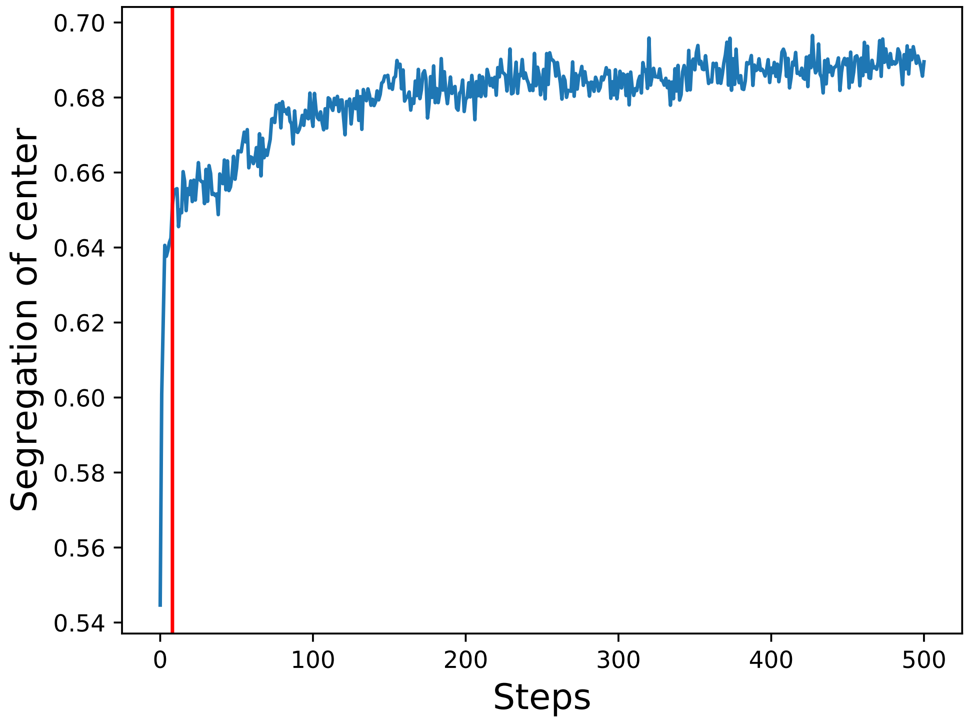

Figure S14: Example of a temporal evolution of the average segregation level within the centre zone during the simulation. The red line indicates the step at which the percentage change calculated respect previous 5 steps is lower than 2%.
